# Isolated Pauci-Immune Pulmonary Capillaritis Associated with Hydrocarbon Inhalation and Marijuana Smoking: An Unusual Case of Severe Hypoxemia

**DOI:** 10.1155/2020/1264859

**Published:** 2020-01-19

**Authors:** Jason S. Oh, Uni Wong, Divyansh Bajaj, Stella E. Hines

**Affiliations:** ^1^Wellstar Health System, Wellstar Pulmonary and Critical Care Medicine, Medical College of Georgia at Augusta University, Department of Medicine, Augusta, GA, USA; ^2^Department of Medicine, Division of Gastroenterology, University of Maryland School of Medicine, Baltimore, MD, USA; ^3^Department of Internal Medicine, Quinnipiac University Frank H. Netter MD School of Medicine/St. Vincent's Medical Center, Bridgeport, CT, USA; ^4^Department of Medicine, Division of Occupational and Environmental Medicine and Division of Pulmonary & Critical Care Medicine, University of Maryland School of Medicine, Baltimore, MD, USA

## Abstract

We present a case report of a patient with Isolated pauci-immune pulmonary capillaritis (IPIPC). A 40-year-old male presented with acute onset severe hypoxemic respiratory failure. He had just returned home from work as a cabinetmaker, where he experienced inhalational exposure to hydrocarbons and solvents, and had smoked a marijuana cigarette. He was hypotensive, and his chest imaging showed bilateral dependent infiltrates. His hypoxemia made little improvement after conventional ventilator support and broad-spectrum antibacterial therapy and he was considered too unstable to tolerate diagnostic bronchoscopy with bronchoalveolar lavage. His laboratory evaluation initially showed microscopic hematuria which later cleared, but other tests including serologic autoimmune assessment were negative, and he did not have any traditional risk factors for vasculitis. A video-assisted thoracoscopic lung biopsy revealed diffuse alveolar hemorrhage with pulmonary capillaritis on histopathology. He was diagnosed with IPIPC and initiated on immunosuppressive therapy. He was soon liberated from mechanical ventilation and improved to hospital discharge. Diffuse alveolar hemorrhage from Goodpasture's Syndrome has manifested following inhalation of hydrocarbons and following smoking. This has not previously been reported with IPIPC. Given the lack of other findings and risk factors, his IPIPC was likely associated with occupational exposures to hydrocarbons as a cabinetmaker compounded by marijuana smoking.

## 1. Introduction

Isolated pauci-immune pulmonary capillaritis (IPIPC) is a rare clinical entity. It is a small vessel vasculitis limited to alveolar capillaries with the absence of systemic manifestations. In most cases, serological testing for antineutrophilic cytoplasmic antibodies (ANCA) is also negative. No specific etiology of the disease has yet, been identified. We present a case of IPIPC temporally associated with inhalation of hydrocarbons as a potential occupational trigger of insult coupled with marijuana smoking.

## 2. Case Report

**Figure 1:**
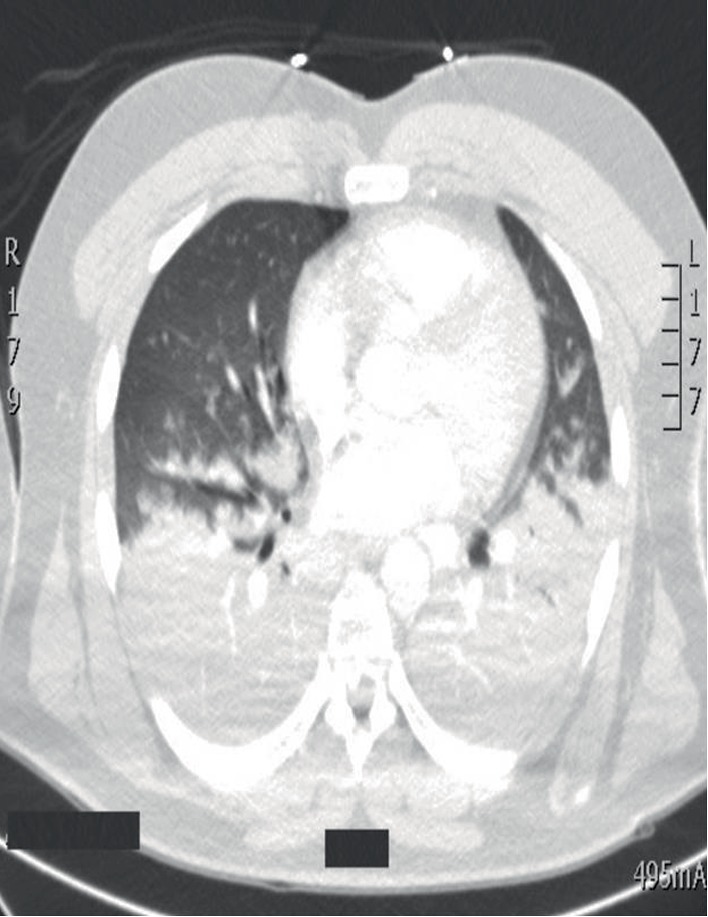
CT chest (axial view) demonstrating bilateral lung infiltrates, more prominent in the lower lobes.

**Figure 2:**
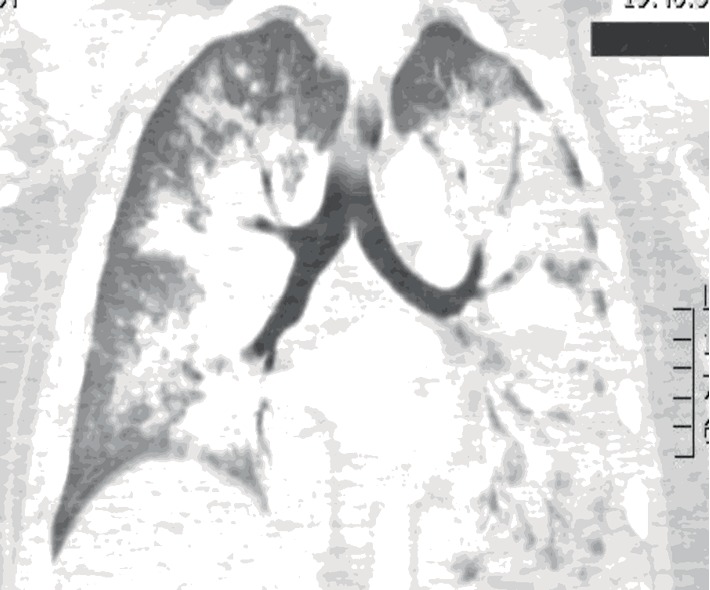
CT chest (coronal view) demonstrating bilateral diffuse lung infiltrates.

A previously healthy 40-year-old Caucasian man was transferred to the intensive care unit with acute hypoxemic respiratory failure (AHRF). He was found unarousable by his wife about 1 hour after arriving home from his job at an industrial cabinet production facility. Emergency medical services brought him to an outside institution where they noted pink frothy secretions on intubation. His white blood cell count was 15,800/mm^3^, hemoglobin 17.4 gm/dL, and platelets 252,000/*μ*L. His bicarbonate was 16.8 mmol/L, blood urea nitrogen (BUN) 15 mg/dL, creatinine (Cr) 1.8 mg/dL and glucose 371 mg/dL. An arterial blood gas (ABG) showed a pH of 6.99, PaCO_2_ 68 mmHg and PaO_2_ of 63 mmHg on an FiO_2_ of 1.0 and PEEP of 10 cm H_2_O. These findings suggested volume depletion with a combined metabolic and respiratory acidemia. Chest computed tomography (CT) angiography was negative for pulmonary embolism but showed bilateral dependent airspace opacities (Figures 1 and 2). Toxicological work-up revealed only tetrahydrocannabinol (THC). He was then transferred to our institution for further management.

Upon arrival, he remained hypoxemic and hypotensive. ABG showed a pH of 7.20, PaCO_2_ of 52 mmHg and a PaO_2_ was 53 mmHg on an FiO_2_ 1.0 and PEEP 15 cm H_2_O. White blood cell count was 11,900/mm^3^, hemoglobin 18.4 gm/dL, platelet 244,000/*μ*L, BUN 13 mg/dL and Cr 1.23 mg/dL with normal electrolytes. Urine analysis (UA) showed >50 red blood cells (RBCs) per high-powered field and 1 + protein without active sediment or casts on microscopy. Liver and coagulation panels were normal. His lactate level was 4.0 mmol/L, later improving to 2.5 mmol/L.

The patient was considered clinically too tenuous to safely tolerate a diagnostic bronchoscopy with bronchoalveolar lavage, given the high FiO_2_ and PEEP requirements. Infectious workup included endotracheal aspirate culture, blood cultures, urine culture, urine legionella antigen and serum cryptococcal antigen. All were negative, and the patient remained afebrile throughout hospitalization.

Electrocardiography did not suggest ischemia and saline-contrasted transthoracic echocardiography demonstrated no intracardiac shunt but suggested pulmonary hypertension with the right atrial pressure >15 mm Hg by inferior vena cava response to the sniff test. Antibacterial therapy consisted of piperacillin/tazobactam, vancomycin and azithromycin.

The differential diagnosis included a vasculitic pulmonary renal syndrome given the AHRF, frothy secretions despite normal cardiac function, and microscopic hematuria. Human immunodeficiency virus screening, antinuclear antibody, ANCA profile, anti-proteinase 3 and anti-myeloperoxidase antibodies were all negative. Hypercoagulability studies with antiphospholipid antibody, anticardiolipin antibody and dilute Russell Viper Venom test were also negative. His microscopic hematuria improved following hydration. The C-reactive protein level was 8.4 mg/dL and erythrocyte sedimentation rate 105 mm/hr.

Video-assisted thoracoscopic surgery (VATS) was performed for lung biopsy on hospital day 3 for severe hypoxemia despite antibiotics and lung protective ventilation and concern for vasculitis. Intraoperative bronchial wash with bacterial and fungal cultures as well as Pneumocystis jirovecii antigen, respiratory viral nucleic acid polymerase chain reaction (PCR) panel and acid-fast bacilli (AFB) smear with culture were negative.

**Figure 3:**
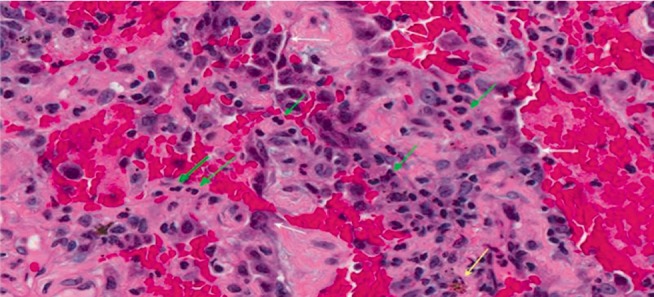
High magnification view showing intra-alveolar hemorrhage, widened septa containing neutrophils (green arrows) and occasional hemosiderin macrophages (yellow arrow). Pneumocytes (white arrows) show reactive changes indicative of acute lung injury (Hematoxylin-eosin ×400).

Histology showed widespread intra-alveolar hemorrhage with organizing injury, hemosiderin-laden macrophages, scattered intra-arterial thrombi and diffuse perivascular neutrophilic infiltrates consistent with capillaritis in all lobes of the right lung. There were no eosinophilic or lymphocytic infiltrates, granulomas, or fibrotic areas. Immunofluorescence for complement and antibodies was also negative. AFB, fungal & bacterial cultures of the biopsy specimen were unremarkable (Figure 3).

With these unexpected results, an occupational medicine consultation was requested for possible exposure-related contributions to our patient's pulmonary vasculitis. Further history revealed that he had not changed clothes upon arrival home directly from work. Within the hour interval between his arrival and unresponsiveness, he had smoked a rolled marijuana cigarette. His workplace manufactured commercial cabinets out of pre-fabricated materials varying from wood products to polymer-based plastics. The patient's tasks included spraying volatile solvents, coatings and lacquers onto the cabinetry. Important components of the aerosolized materials included methyl acetate, methoxymethane, hexane, 2-methylpropane and propane. He did not work with any acid anhydrides. He sometimes, wore a paper, non-fit-tested mask during his work. The patient was chronically exposed to these aerosolized chemical agents for at least several years. He also used to smoke marijuana intermittently a few times every week, for several years.

**Figure 4:**
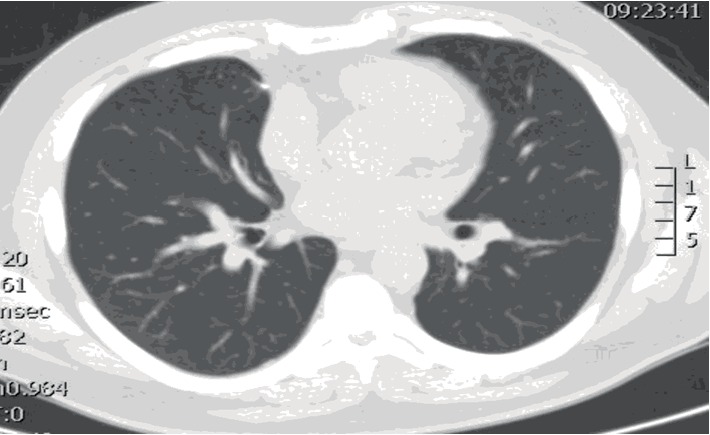
CT chest (axial view) demonstrating complete resolution of lung infiltrates, two months post discharge from the hospital.

**Figure 5:**
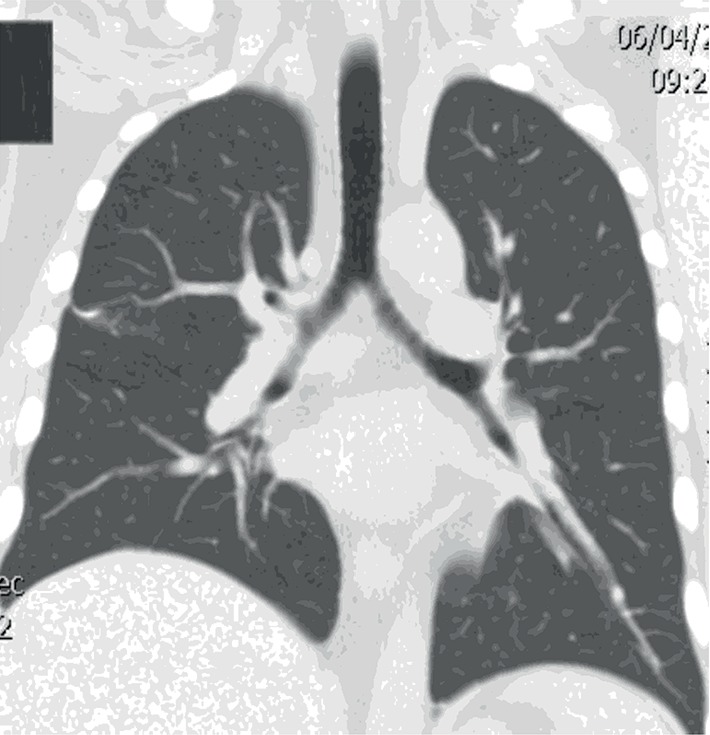
Coronal CT chest at lung window showing complete resolution of infiltrates, two months post discharge from the hospital.

Given the dense pulmonary vasculitis with severe AHRF, negative immune studies, and lack of evidence for extra-pulmonary vasculitis, the patient was diagnosed with isolated pauci-immune pulmonary capillaritis. Intravenous cyclophosphamide at 750 mg/m^2^ and intravenous methylprednisolone 1,000 mg per day were initiated on day 8 of mechanical ventilation with rapid improvement in respiratory function and ventilator liberation the next day. CT imaging two months later revealed radiographic resolution of his disease (Figures 4 and 5).

## 3. Discussion

Diffuse alveolar hemorrhage (DAH) manifests with red blood cells (RBCs) scattered throughout the intra-alveolar space. The classic presentation of hemoptysis, bilateral diffuse infiltrates on chest radiograph, and anemia is uncommon, thus, making the diagnosis challenging. Bronchoscopy with bronchoalveolar lavage (BAL) showing increasing RBC counts on serial aliquots and the presence of hemosiderin [1] is diagnostic of DAH. Once confirmed, a good history and physical (H&P) with laboratory evaluation often reveals the etiology among many potential causes: infectious [2] precipitators of diffused alveolar damage, coagulation disorders, idiopathic pulmonary hemosiderosis and immune-related disorders such as the vasculitides. The etiology is important, as treatment can include corticosteroids, cytotoxic agents or immune-modulators.

Pulmonary capillaritis is a subset of vasculitis with damage to the alveolar microcirculation [1]. Renal or lung biopsy should be performed if the H&P with serologic testing is nondiagnostic when the BAL shows DAH. Renal biopsy is typically performed when suspecting a pulmonary-renal syndrome, such as granulomatosis with polyangiitis or Goodpasture's syndrome, as it is usually considered less invasive than lung biopsy [3]. Lung histological findings include intra-alveolar RBCs, erythrophagocytosis, hemosiderin-laden macrophages and neutrophilic infiltration of the interalveolar interstitium.

Although aspiration pneumonia was considered initially as a cause of his AHRF, the patient's presentation seemed inconsistent with a septic etiology given his lack of fever, his cool extremities and negative cultures. DAH from vasculitis seemed possible, but still unlikely given his lack of other systemic manifestations, negative serologies and abrupt onset of symptoms.

The initial high ventilator settings, his profession as a cabinetmaker and the lack of abnormal noninvasive diagnostic clues concerned us for a vasculitis or atypical pneumonitis that would be best diagnosed via surgical biopsy, instead of bronchoscopy with BAL. Renal biopsy was considered but felt to be of low yield given the normalization of his urinalysis and renal function without specific intervention.

Our suspicions were confirmed on VATS biopsy which showed DAH with pulmonary capillaritis. IPIPC is a rare entity in medical literature. In 1997, Jennings et al. described pauci-immune pulmonary capillaritis in eight out of twenty-nine patients with DAH and biopsy proven pulmonary capillaritis. We performed a search on PubMed and MEDLINE on key words of “pauci immune pulmonary capillaritis” and “diffuse alveolar hemorrhage” and identified eight case reports where pauci-immune pulmonary capillaritis resulted in DAH [4–11] and one where there was no evidence of DAH, only neutrophils in the interalveolar interstitium on histology [12].

Inhalation exposures have been associated with pulmonary hemorrhage syndromes [13, 14]. These include hydrocarbon exposures and smoking with pulmonary hemorrhage in Goodpasture's syndrome [15, 16]. A direct causal relationship has not yet been found; however, numerous studies demonstrate the association [17–27]. Direct damage to the alveolar-capillary interface is hypothesized to expose the vulnerable basement membrane to immune reactivity and development of Goodpasture's syndrome in susceptible individuals [28]. The culprit antigen is found in the collagen type IV component of the basement membrane. Exposure to hydrocarbons and smoking is thought to cause a conformational shift in the quaternary structure of one of the collagen hexamers [*α* 345NC1] to expose the antigen(s) [18].

A toxicological study demonstrated that systemic administration of hydrocarbon oil can induce vasculitis and DAH in mice within 2 weeks of exposure in a dose-dependent fashion [29]. The pathogenesis of DAH resulting from hydrocarbon oil was attributed to the infiltration of lung parenchyma by B1B cells, a specific subset of B cells. B1B cells act by producing large amounts of IgM antibody which has been implicated in triggering a complement-mediated inflammatory response. DAH from vasculitis is increasingly being treated with the B cell-depleting rituximab, which may be an optimal therapy for hydrocarbon-associated DAH [30]. The authors also found that oropharyngeal aspiration of even very low doses of hydrocarbon oil led to significant lung inflammation and minimal hemorrhage in mice. This may share similarities with human opportunities for hydrocarbon inhalational exposure.

While this patient did not meet criteria for Goodpasture's syndrome, perhaps there was a similar mechanism of injury. His lack of traditional risk factors for vasculitis or connective tissue disease makes an exposure injury more suspect, especially given the occupational inhalation exposure to volatile hydrocarbons followed by marijuana smoking. He had worked with several different volatile products.

The spray adhesive used to seal wood paneling to a polymer coating was composed of methyl acetate (25–35%), methoxymethane (15–25%), hexane (10–20%), 2-methylpropane (5–10%), and propane (5–10%) [31]. Our patient did not wear a respiratory protective device, but rather a non-fit-tested paper mask during work. This would not have prevented inhalational exposure to a gas or vapor. We hypothesize that his hydrocarbon exposure, combined with smoking, could have damaged his alveolar-capillary interface via a mechanism like that of Goodpasture's syndrome.

Jennings et al. reported that three of their eight patients with pauci-immune pulmonary capillaritis were also marijuana smokers [32]. The association between marijuana use and DAH has been demonstrated in several recent case reports [33–36]. A few studies have also implicated marijuana use in causing airway inflammation and parenchymal injury which could further contribute to the occurrence of vasculitis and DAH [37, 38]. Alternatively, the combustion of a previously inhaled hydrocarbon as one smokes tobacco or marijuana may serve as a trigger for vascular injury.

We hypothesize that our patient sustained small vessel injury from hydrocarbon exposure combined with marijuana smoking, resulting in IPIPC and severe hypoxemic respiratory failure. This potential association between hydrocarbon inhalational exposure and IPIPC is the first case that we know of in the literature. Additional studies further exploring this possible association are needed. As availability of recreational and medical marijuana continues to rise among working adults, recognition of this association is also needed in the medical community.
